# Selecting an Effective Entropy Estimator for Short Sequences of Bits and Bytes with Maximum Entropy

**DOI:** 10.3390/e23050561

**Published:** 2021-04-30

**Authors:** Lianet Contreras Rodríguez, Evaristo José Madarro-Capó, Carlos Miguel Legón-Pérez, Omar Rojas, Guillermo Sosa-Gómez

**Affiliations:** 1 Facultad de Matemática y Computación, Instituto de Criptografía, Universidad de la Habana, Habana 10400, Cuba; lianecontreras@gmail.com (L.C.R.); ejmcapo@gmail.com (E.J.M.-C.); clegon58@gmail.com (C.M.L.-P.); 2 Facultad de Ciencias Económicas y Empresariales, Universidad Panamericana, Álvaro del Portillo 49, Zapopan, Jalisco 45010, Mexico; gsosag@up.edu.mx

**Keywords:** entropy, estimation, cryptography, randomness, undersample

## Abstract

Entropy makes it possible to measure the uncertainty about an information source from the distribution of its output symbols. It is known that the maximum Shannon’s entropy of a discrete source of information is reached when its symbols follow a Uniform distribution. In cryptography, these sources have great applications since they allow for the highest security standards to be reached. In this work, the most effective estimator is selected to estimate entropy in short samples of bytes and bits with maximum entropy. For this, 18 estimators were compared. Results concerning the comparisons published in the literature between these estimators are discussed. The most suitable estimator is determined experimentally, based on its bias, the mean square error short samples of bytes and bits.

## 1. Introduction

Entropy allows the measurement of the uncertainty about an information source from the distribution of its output symbols [1]. The Shannon’s entropy of a discrete source of information will be maximum if the source transmits symbols that follow a Uniform distribution; that is, the highest level of uncertainty.

In most cases, the data for the measurements are obtained but the distribution is unknown, so it is necessary to estimate the parameters. Even knowing the data distribution, it is necessary to determine which is the best performing entropy estimator since there is no single ideal estimator for all the distributions that may occur. On the other hand, on many situations, there are not enough samples with sufficient sizes compared to the source alphabet, in certain scenarios called *undersample regime*, where many estimators underestimate the real entropy [2,3]. It is well known that there is not an unbiased estimator [4], and the convergence rate of a consistent estimator can be arbitrarily slow [5]. However, there are many estimators, but deciding which estimator to use depending on the scenario turns out to be a practical problem [6].

Estimating a random variable’s entropy is of great importance and with many applications, having received much attention in recent years [7,8,9,10,11]. Most of the known estimators are generally applied for the estimation of mutual information [12], but in this scenario entropy estimators will perform similarly, and thus the selection between different estimators is less of an issue. However, entropy has great application in cryptography as a vital tool for designing and analyzing encryption methods [13,14,15,16,17]. Generally, in cryptography, one usually deals with large sample volumes [18,19,20,21,22], so the estimation of entropy is often useless, as a very similar behavior is obtained for all estimators. However, it is possible to find scenarios in cryptography where the estimation of entropy is vital since it works with smaller samples [23,24]. In these cases, it is necessary to have the estimator with the highest convergence rate and the lowest mean square error to increase the result’s precision.

There is a wide variety of entropy estimators [3,5,25,26,27,28,29,30,31,32,33,34,35,36,37,38,39,40,41,42,43,44,45] tested in dissimilar scenarios. However, it is not easy to find results of applying or selecting entropy estimators in samples uniformly distributed composed of bytes and bits. In this work, the most suitable method to estimate entropy in short samples of bytes and bits uniformly distributed is determined experimentally from the bias and mean square error characteristics. For this, 18 estimators reported in the literature were compared, and the results are discussed concerning the literature found. The structure of the paper is as follows: Section 2 presents some preliminaries about entropy and comparison criteria for the estimators; Section 3 discusses some of the entropy estimators from the literature; Section 4 presents the main results, which have to do with the selection of the entropy estimators; finally, Section 5 presents some conclusions and possible future lines of work.

## 2. Preliminaries

### 2.1. Shannon Entropy

Shannon’s entropy is a measure of a random variable’s uncertainty. Let *X* be a discrete random variable with the alphabet *K* and the probability function $p_{x}=Pr\left\{X=x\right\}$, x∈K, then we have the following

**Definition** **1.** *The entropy $H\left(X\right)$ of a discrete random variable X is defined as [1]:*

$$H\left(X\right)=-\sum_{x\in K}^{}p_{x}\;\log_{2}\;p_{x}.$$

A discrete source (*source with a finite alphabet*) with alphabet *K* reaches its maximum entropy $H_{\max}$ if its output symbols follow a uniform distribution. In this case, any discrete random variable on this alphabet has an entropy no greater than $\log_{2}\;\left|K\right|$ [1]. On the other hand, the lowest value of the entropy $H_{\min}=0$ is reached when, for some x∈K one has that $p_{x}=1$. The maximum possible value for the entropy of a discrete random variable with *k* different values (where |K|=k) $H_{\max}=\log_{2}\;k$ is reached when the random variable has a uniform probability distribution. Thus, since the objective is to select an effective estimator of entropy for sequences of bytes and bits with maximum entropy, the selected estimator’s results will be compared with the expected value of entropy for uniformly distributed samples, $H_{\max}$.

In [46], a lower bound on the convergence rate of an optimal estimator is provided. In there, they theoretically demonstrated that with a sample of size $k/\log_{2}k$ the entropy can be estimated, where *k* is the alphabet’s size. The undersample regime happens when the size *n* of the samples is less than the size *k* of the alphabet and is denoted: n<k [47]. The size of the alphabet for the case of byte samples is 256 and for the case of samples of 2-bit. Thus, the entropy value can vary from $H_{\min}=0$ to $H_{\max}=8$ and from $H_{\min}=0$ to $H_{\max}=1$, for byte and bit samples, respectively. This work focuses on samples with sizes greater than or equal to n=8 bytes and bits, respectively. In the case of bit samples, it was decided to work with 8 bits because this constitutes the smallest value of *n* most frequent in current encryption algorithms.

### 2.2. Comparison Criterion between the Estimators of H

As mentioned before, it is well known that there is not an unbiased estimator [4] and of minimum variance, while the convergence rate of a consistent estimators can be arbitrarily slow [5]. Two well-known statistical tools of estimation theory will be used to experimentally evaluate the selected estimators, bias and mean square error [48]. The bias $S\left(\hat{H}\right)$ of an estimator $\hat{H}$ measures the deviation of the estimate with respect to the expected real value and is calculated from the difference $S\left(\hat{H}\right)=E\left(H\right)-\hat{H}$, where $E\left(\hat{H}\right)$ is the expected value of $\hat{H}.$ In our scenario, we will work with uniformly distributed samples of bytes and bits so the data follows a uniform distribution and we have that $E\left(\hat{H}\right)=H_{\max}$.

The mean square error (MSE) of an estimator measures the average of the squared errors, that is, the difference between the estimator and what is estimated. The difference occurs by two possible reasons—by randomness or because the estimator does not consider the information that could produce a more accurate estimate. The MSE is a measure to determine the quality of an estimator with a non-negative value. If the value of the MSE is close to zero, then it implies that the quality of the estimator is better. For an unbiased estimator, the MSE is the variance of the estimator. That is, if the variance of an estimator is smaller among all the estimators, then it will be considered as the best unbiased estimator. The MSE is equal to the sum of the variance and the square of the estimator bias,

$$\mathrm{MSE}\left(\hat{H}\right)=Var\left(\hat{H}\right)+S{\left(\hat{H}\right)}^{2}.$$

Thus, the MSE assesses an estimator’s quality in terms of its variance and the degree of bias.

## 3. Entropy Estimators

Various entropy estimators have been proposed in the literature with the aim of approaching its theoretical value in practice. This section briefly describes 18 entropy estimators present in several works published in the literature. The empirical approach estimates entropy from the observed individual and joint frequencies for each bit. The maximum likelihood estimator (ML) of entropy is defined as follows

$${\hat{H}}^{ML}=-\sum_{x\in K}^{\;}{\hat{p}}_{x}^{ML}\log_{2}{\hat{p}}_{x}^{ML}.$$

The probability of appearance in the sample with respect to the observed frequencies $y_{x}$ for each x∈K is specified as ${\hat{p}}_{x}^{ML}=y_{x}/n$, where *n* is the sample size. Table 1 describes the others 17 entropy estimators that will be used.

Apart from the entropy estimators described, there are many others in the literature, such as the KDE [36], KNN [39], B-Spline [35], Edqeworth [37], Polynomial [38] and the Bayesian PYM and DPM [6]. This shows that entropy is a very well studied information theory tool in the current literature.

### 3.1. Theoretical Approximations between Estimators of Entropy

It is well known that there are relationships between various estimators depending on the scenarios that are being worked on. These can coincide based on their formulation and on the values of their parameters, such as the probability distribution, the size of the alphabet, or eigenvalues used in the formulation of the estimator. There is a relation between the SHR and Bayes estimators [32]. Using $t_{k}=a_{k}/A$ and $\lambda =A/\left(n+A\right)$, then ${\hat{p}}_{x}^{SHR}={\hat{p}}_{x}^{Bayes}$. This show that the SHR estimator is an empirical Bayes estimator based on the $t_{k}$ and λ selected values. For a choice with no shrinkage ( λ=0 ), ${\hat{p}}_{x}^{SHR}=$${\hat{p}}_{x}^{ML}$ is obtained and ML estimator is achieved. On the other hand, if  $a_{k}=0$ is selected for Bayes estimator, then ${\hat{p}}_{x}^{Bayes}={\hat{p}}_{x}^{ML}$ and again ML estimator is achieved. In addition, for bit samples the SG estimator must coincide with the JEFF estimator because in this case k=2. In [50], the Zhang estimator was classified within the family of entropy estimators originally introduced in [42]. Also, in the asymptotic regime $y_{x}\gg 1$, the estimator given for the choice ξ=1 within the family of entropy estimators in [42] leads to the MM estimator using the asymptotic relation $\psi \left(x\right)∼\log\left(x\right)-1/2x$. The BUB estimator leads exactly to a version of the Miller-Madow correction and gives another angle on why this correction fails in the N∼m regime [4]. In this perspective, the MLE estimator can be expressed as

$${\hat{H}}^{MLE}=-\sum_{i=0}^{n\;}a_{i}h_{i},$$

where $a_{i}=-\frac{i}{n}\log\frac{i}{n}.$

### 3.2. Previous Work on Comparison of Entropy Estimators

Many of the entropy estimators were thought for specific contexts, so there has been a tendency to compare which estimator would be the most suitable to be used in each scenario. The following reports present various comparisons between entropy estimators described in the literature. This allows a vision of the best behavior estimators even when the used scenarios do not coincide with those of this paper. In [4], four different estimators were analyzed from various statistical points of view, such as the central limit theorem’s consistency, bias, and variance. In samples of real and simulated spike trains, it was obtained that the behavior of the estimation with the BUB approach is better than with the use of the ML, MM, and Jackknife methods.

In [32], nine estimators (MM, ML, NSB, CS, Shrink, MIN, SG, JEFF and LAP) on the Dirichlet distribution, with different structures and values of the parameter *a*, and the Zipf-type power law were compared. In summary, the three best-performing estimators are the NSB, CS and Shrink estimators, with very similar behavior in the scenarios presented. However, in his simulations, he emphasizes that the Shrink estimator is the only one that estimates probabilities with high precision for the use of Shannon entropy, even for small samples. On the other hand, the rest of the estimators can be considered with similar behavior in several scenarios. Specifying that the Bayesian estimators can have better or worse behavior than the ML estimator, depends on selecting the prior and the sample scenario but without a marked difference. In the case of Bayesian estimators, there is no general agreement on which allocation of *a* is better a priori. However, as discussed in [32], choosing an inappropriate *a* can cause the resulting estimator to perform worse than the ML estimator, influencing the practical results.

In [33], six entropy estimators—ML, MM, Jackknifed, CS, BUB and Unseen—were compared. The samples were drawn from six different classes of distributions—the uniform distribution, a mixture of two uniform distributions; the Zipf distribution, which is commonly used to model naturally occurring “power law” distributions, particularly in natural language processing, a modified Zipf distribution with 0.6 power-law exponent, the geometric distribution, and finally, a uniform mixture of Geometric and Zipf. For each distribution, three configurations of the size *k* of the alphabet were considered: 1000, 10,000, and 100,000. The sample size, *n*, varied in the interval $\left[k0.6,k1.25\right]$. It was found, as a result, that the three best-performing estimators were the Unseen, the CS, and the BUB, where the Unseen estimator being ranked above these three estimators. Specifically for larger sample sizes and in samples from the Zipf distribution with respect to the CS estimator and for the uniform and Zipf distributions with respect to the BUB estimator. The Unseen estimator performance on all classes of distributions is significant since the algorithm proposed for its computation is designed to compute a representation of the distributions rather than specifically adapted to estimate entropy.

In [51], the effects of entropy estimation on mutual information and data discretization in the inference of gene regulatory networks were investigated. Here, they evaluated the performance of the MM, ML, SG, and Shrink estimators with three different discretization approaches (equal frequency, equal amplitude, equal global amplitude) and stated that changing the discretization approach influences the result more than changing the estimators. They indicated that using the MM estimator with the discretizations of equal amplitude and equal global amplitude achieves the best inference result. Samples of size 50, 100, 200, 500, 1000 were used in the experiments. In [34], some experiments were run with 6 estimators ML, Jackknife, NSB, Zhang, and two corrections of the Zhang estimator (correction proposed in [33]) and (Jackknife correction). The comparison of the estimators was made in three categories: finite alphabets with known cardinalities, finite alphabets with unknown cardinalities, and infinite alphabets. For each case in the simulation, the bias reported was based on the average of 5000 simulated samples. The sample size varied from ten to several hundred. The jackknifing was done using all subsamples of sizes *m*, where 5<m<n. The experiments were carried out on six distributions, Zipf, Poisson, Gauss and variations of these.

The ML estimator is dominated by the Zhang estimator and the Jackknife estimator by the correction of the Zhang estimator with the Jackknife. The NSB estimator has a better behavior but very similar to the Zhang estimator with the Jackknife correction. It is highlighted that in this case the Jackknife correction provides a better performance to the Zhang estimator than with its proposed correction. It is reported that the NSB has a tendency to overestimate the entropy value and they expose it in cases with the Poisson distribution and the Gaussian distribution with certain characteristics. In [43], five estimators (Jackknife, Zhang with correction, CS, CWJ and GSB) were compared, modeling species abundance distributions. The number of species was fixed to be 100, 500 or 1000 and seven species abundance models were considered, a homogeneous model, the log-normal model, the Zipf–Mandelbrot model, the broken-stick model, power-decay model, Poisson model and exponential-decay model. For each fixed model, considered a range of sample sizes (n=25−500 in an increment of 25 if S<500, and n=50−1000 in an increment of 50 if S≥500). For each combination of abundance model and sample size, 5000 simulated data sets were generated from the model. As results, the GSB, Jackknife and Zhang’s estimators are biased downwards and the three estimators have similar trends. The GSB estimator is less biased than the Jackknife, which is less biased than the Zhang’s estimator. The RMSE shows the same pattern. On the other hand, CWJ estimator in nearly all cases is better than the GSB, Jackknife and Zhang’s estimators based on the criteria of bias and RMSE. The CS estimator generally has smaller bias and RMSE than the GSB estimator.

The results described in this section corroborate the problem of selecting the ideal entropy estimator for a particular practical case. In summary, these results highlight how factors such as sample size, sample distribution, alphabet size, sample discretization method, and optimal selection of distribution parameters can influence the behavior of the estimators or the optimal selection of parameters in the calculation of the estimator. The comparison of entropy estimators presented in this work differs from the previous ones in several respects. First, 18 estimators are compared, a figure much higher than in previous studies. Second, it is limited to discrete samples, specifically of bits and bytes from the uniform distribution, which are of great cryptographic importance, so discretization is unnecessary. Third, the greatest interest is in the short sample regime for byte samples when the sample size is less than the alphabet’s size. However, for the bit case, it has also experimented with short samples. Fourth, of the 17 compared estimators, 15 are present in previous works, while 2 (CDM and BN) provide new comparisons. Fifth, an experimental correlation study between entropy estimators in this scenario is presented for the first time.

## 4. Selecting an Effective Entropy Estimator through Experimental Evaluation

In this section, the 18 estimators were applied to short uniformly distributed samples of bytes and bits obtained using the Linux urandom random number generator [52]. Then, the results obtained through their bias and their mean square error were compared, illustrating the behavior of their characteristics using some plots. Also, the selection of the most effective estimator as a result of the comparison made is discussed. To carry out the experiments, 1000 samples of uniformly distributed sequences of bytes and bits were generated for each of the sizes 8, 16, 32, 64, 128, 256, 512, 1024, 2048, 4096, 8192, and 16,384. In [53], it shows how the output sequences of random number generators must follow a uniform distribution. Thus, the values of the estimators will be compared with the expected theoretical value for this distribution (for uniformly distributed samples in bytes, k=256 and $H_{max}=8$, and for the case of uniformly distributed bit samples k=2 and $H_{max}=1$). Cases in the regime of short samples were analyzed, and situations when the sample size exceeds the size of the alphabet, that is, n>k, managing to visualize the convergence of the estimators.

### 4.1. Implementation of Entropy Estimators

The estimators ML, MM, JEF, LAP, SG, MIN, CS, SHR are available in the R software package Entropy [53]. The entropy function of that package allows estimating entropy from observed counts. The Zhang estimator’s estimates were made with the EntropyEstimation package [54] of the R software. The Matlab implementation of the BUB estimator provided by the author [4] was used, and its numerical adjustment parameters were left. Likewise, for the Unseen estimator, the author’s Matlab implementation in [33] was used. For the NSB estimator, the Python implementation proposed in [55] was used. The SHU, UnveilJ, GSB, CWJ, and BON estimators can be found in the package Entropart [56] for R. While the CDM estimator is part of the CDMEntropy project implemented in Matlab [57].

### 4.2. Analysis of Bias between Estimators

**Figure 1:**
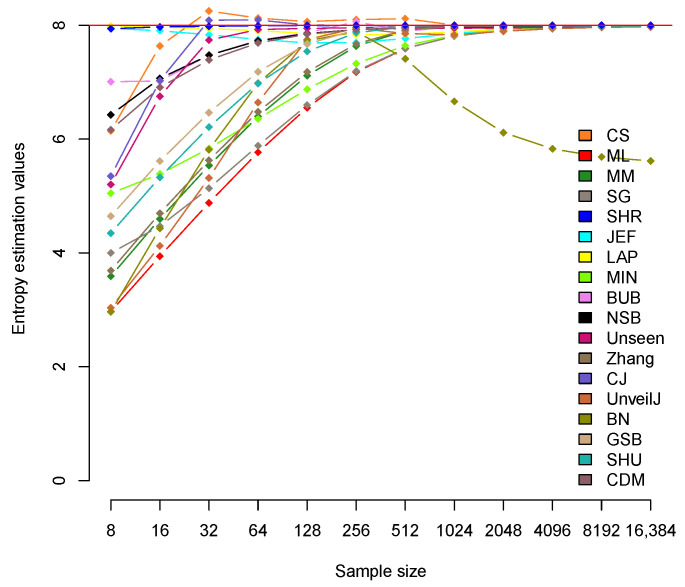
Behavior of the estimated mean of the 18 entropy estimators for different uniformly distributed sample sizes of bytes.

In this section, the bias behavior of the 18 estimators is analyzed. Figure 1 shows the behavior of the estimated mean of the estimators for each sample size for uniformly distributed samples of bytes. The straight red line represents the expected theoretical value, that is, $H_{max}=8$.

The behavior of the 18 estimators in the uniformly distributed samples of bytes is similar in terms of convergence towards the expected theoretical value. However, there are differences between them in terms of the speed at which they converge. When the size *n* of the samples is less than the alphabet’s size k=256, there is a deviation in many of the estimates from the expected theoretical value. Figure 1 shows three estimators (SHR, LAP, JEF) that are considerably close, when n<k, to the expected theoretical value $H_{max}=8$. In this group, the SHR estimator stands out for reaching values very close to $H_{max}=8$ for all sample sizes, as corresponds to a uniformly distributed sample with a uniform distribution. Table 2 shows the average value of $\hat{H}$ for each estimator in all the sizes used. The LAP and JEF estimators show values very close to $H_{max}$ up to n=32, however, for n=256, their value decreases a little.

The rest of the estimators show a similar trend towards the expected $H_{max}$ value. However, the behavior of the second group of estimators (CS, CJ, BUB, NSB, Unseen, CDM) stands out in terms of the speed of convergence towards $H_{max}$. On the other hand, the BN estimator’s unusual behavior is highlighted from n>k=256, coincidentally the size of the alphabet, which confirms that the estimator was constructed for short samples [40]. Furthermore, the CS and CJ estimator show a tendency to overestimate the expected theoretical value in some sample sizes. Starting from n=2048 approximately, the values of all estimators adjust to the expected theoretical value $H_{max}=8$. Table 3 shows the difference ($H_{max}-\hat{H}$) that represents an estimate of the bias in each of the estimators for byte samples. This measure gives the deviation of the expected value of an estimator concerning the real value.

**Figure 2:**
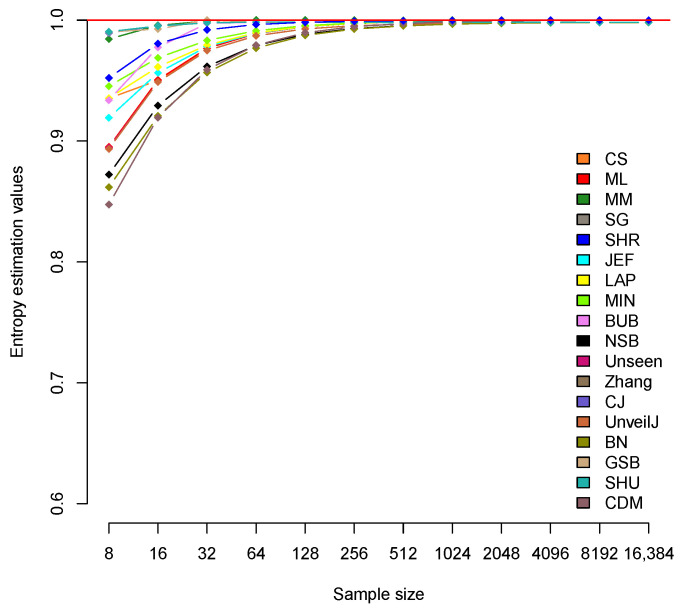
Behavior of the estimated mean of the 18 entropy estimators for different uniformly distributed sample sizes of bits.

Table 3 confirms the SHR estimator as the one with the best performance in this scenario, with almost zero bias for all sizes. In bit samples, a very similar convergence speed is observed for all estimators (see Figure 2). However, a group of estimators stands out, the cases of Zhang, CJ, GSB, SHU, and MM, with values very close to the expected value and faster convergence to it.

To accompany Figure 2 in the visual description of each estimator’s behavior, Table 4 presents the average $\hat{H}$ value of each of the estimators for all sample sizes.

This group is followed by the SHR and BUB estimators, highlighting the BUB estimator with a very fast convergence towards $H_{max}$. Approximately, starting at n=512, the values of all estimators adjust to the expected theoretical value $H_{max}=1$. Table 5 shows the difference ($H_{max}-\hat{H}$) that represents an estimate of the bias in each of the estimators for bit samples.

Table 5 shows the good behavior of all estimators in this scenario. Unlike byte samples in this case, as mentioned above, the estimators show very similar behavior. It is known that the estimated mean can hide behaviors. For this reason, it is necessary to perform an analysis of the variance of these estimators to confirm that the average value of $\hat{H}$ correctly describes the behavior of the estimator in each scenario.

**Figure 3:**
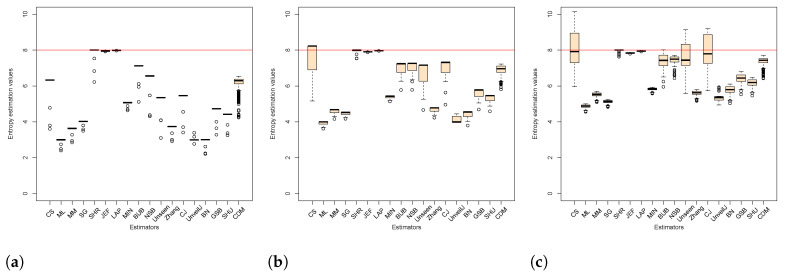

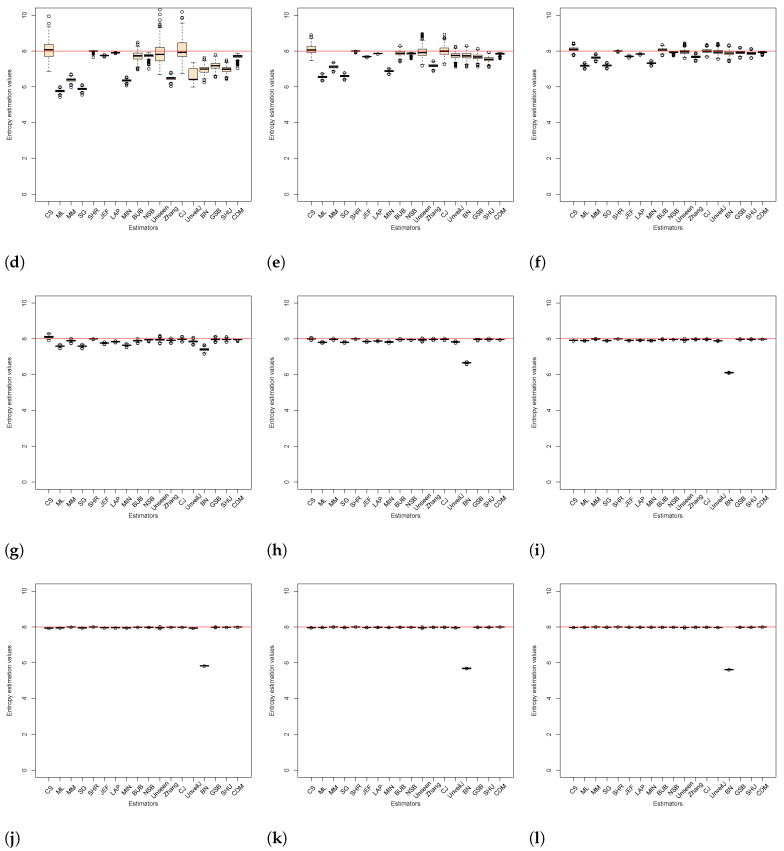
Behavior of the mean and observed variance of the estimators in byte samples using box plots for all sizes. Figure 3 (**a**) corresponds to the sample size n=8, so on until Figure 3 (**l**) for n = 16,384.

**Figure 4:**
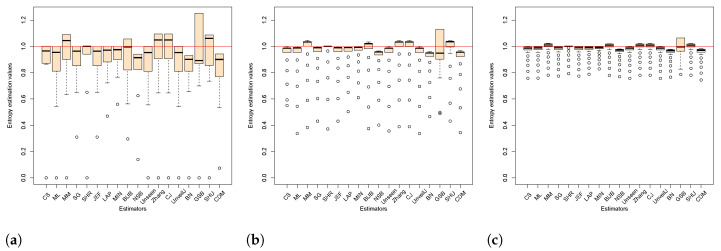

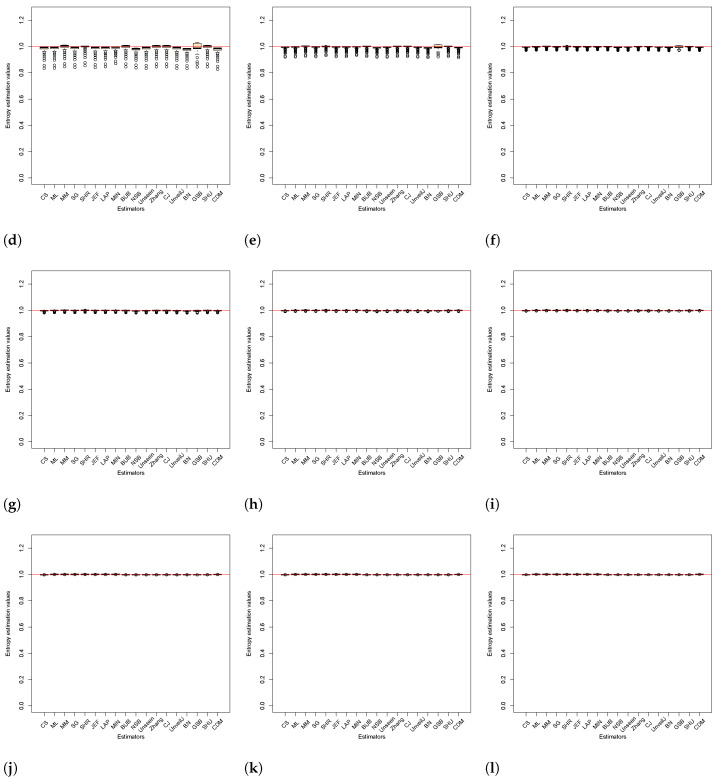
Behavior of the mean and the observed variance of the estimators in bit samples using box plots for all sizes. Figure 4 (**a**) corresponds to the sample size n=8, so on until Figure 4 (**l**) for n = 16,384.

Figure 3 and Figure 4 show the dispersion of the estimated values in the samples of uniformly distributed sequences of bytes and bits, respectively, for each of the chosen sizes. Figure 3 confirms the overestimation of the estimators CS and CJ expected theoretical value for various sizes of *n* (see Figure 3c–e). Furthermore, it shows how the Unseen estimator also has some over estimated values of $\hat{H}$ that are not visible through the average value of $\hat{H}$. Figure 4 shows how for bit samples, the estimators have greater variance. The SHR estimator with the lowest variance and with a concentration of its values closer to $H_{max}$ stands out. On the contrary, the GSB estimator is highlighted as the highest variance with a very unstable dispersion. Although this estimator performs well on average, the variance shows a considerable dispersion of its values.

### 4.3. Comparison of Estimators in Terms of Mean Square Error

**Figure 5:**
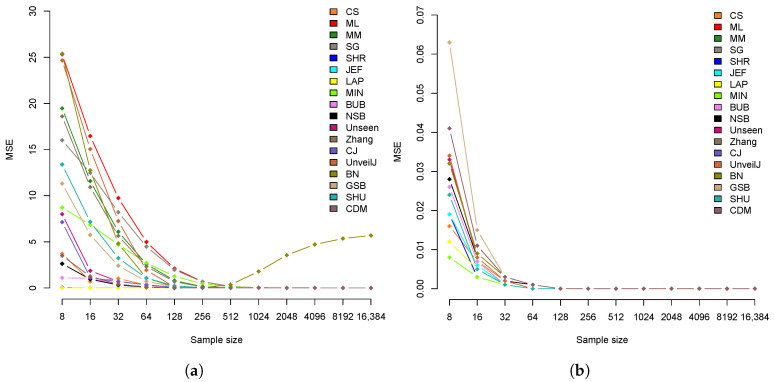
Mean Square Error of Entropy Estimates in Byte (**a**) and Bit (**b**) Samples.

Another of the most used tools to measure an estimator’s quality is the mean square error (MSE). Figure 5 illustrates the estimate’s mean square error in uniformly distributed samples of bytes and bits for each size.

Figure 5a shows a group of seven estimators (LAP, JEF, BUB, NSB, SHR, CS and CDM) with a greater tendency towards MSE=0. Table 6 shows the MSE values for each of the sample sizes.

The SHR estimator has the best behavior, with an MSE very close to 0 for all sizes. This result establishes the SHR estimator as the most suitable, lowest bias, and lowest MSE for uniformly distributed samples of bytes. On the other hand, in Figure 5b, a clear difference is not observed, but 5 estimators can be distinguished (LAP, JEF, CS, MIN, SHR) with better convergence (see Table 7).

It is more difficult to select a suitable single estimator in bit samples since all estimators show good results in terms of bias and MSE value. However, the MM and SHR estimators stand out in both. Therefore, in this scenario, the use of either of these two estimators is suggested, specifying that MM has less bias and SHR has less MSE. In this case, the estimators have similar efficiency. It would be of interest to compare its efficiency since looking for a balance between effectiveness and efficiency, it would be possible to define which estimator to select.

### 4.4. Correlation between Estimators of Entropy Using Bias

**Figure 6:**
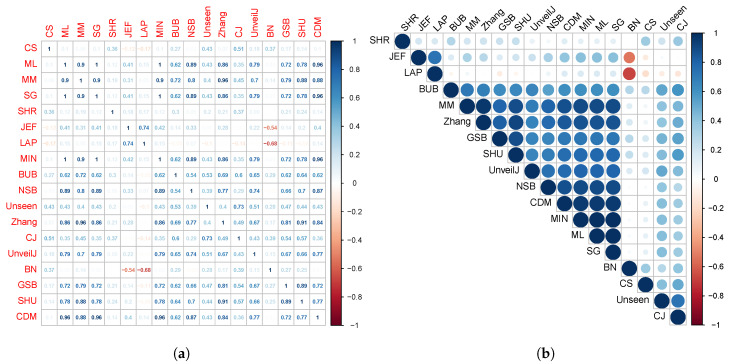
Correlation matrix between the estimators for random samples of bytes. The correlation is represented based on its numerical value (**a**) and the intensity of the color (**b**).

Figure 1 suggests a trend or approach in the distribution of the estimated means of various estimators, which could suggest the existence of a correlation between them. Thus, in this section, the correlation between the estimators will be measured using Kendall’s correlation coefficient. In Figure 6, the Kendall correlation coefficient values between the estimators for random samples of bytes can be observed.

The size of the circles and the intensity of the color are in correspondence with the correlation value existing between the estimators. From the results shown in Figure 6, groups of estimators with higher correlation can be analytically determined. Considering the values of Kendall’s correlation coefficient (CCK) [58], the following four groups are obtained, grouping them by having a correlation value greater than 0.6 or less than −0.6.

1. { JEF, LAP, BN},
2. { MM, BUB, ML, SG, MIN, Zhang, NSB, CDM, UnveilJ, GSB, SHU},
3. { CS, Unseen, CJ}.
4. { SHR}.

**Figure 7:**
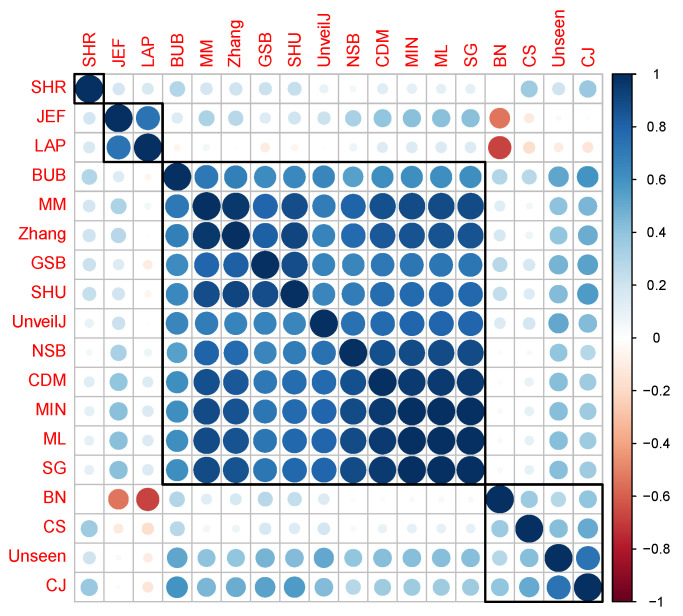
Clustering of estimators in uniformly distributed byte samples using the full-link agglomerative method.

The third and fourth group’s estimators stand out for their rapid convergence in byte samples; therefore, it is suggested in terms of bias to use one of these estimators in this scenario. However, the SHR estimator stands out for its excellent behavior for all sample sizes. This grouping of the estimators was corroborated using the hierarchical grouping method to determine the highest correlation groups. Hierarchical grouping algorithms are used for grouping patterns whose internal organization is unknown; that is, there is no knowledge about the class label to which they belong. In data mining, these data groups are called clusters. Figure 7 shows the clustering performed using the full link agglomerative method [59,60] with the distance based on the correlation, in this case, the Kendall correlation.

**Figure 8:**
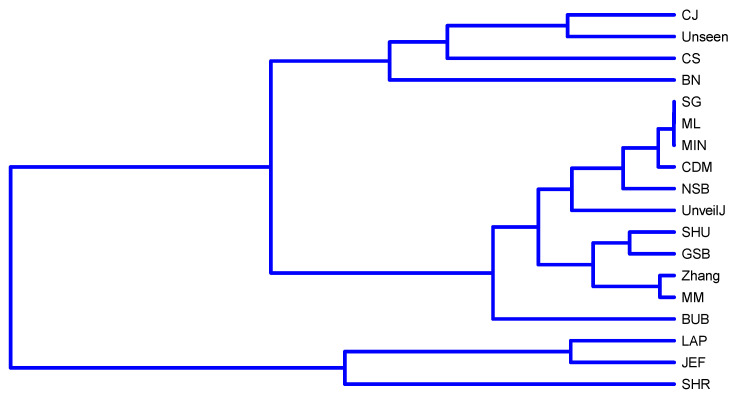
Dendrogram corresponding to the hierarchical grouping of the estimators for uniformly distributed samples of bytes.

The BN estimator is in a different group, possibly because its correlation with the JEF and LAP estimators is in the opposite direction, that is, negative. Furthermore, some of these groups can be divided consecutively into subsets such that the value of the coefficient has less variation within each subset. Figure 8 shows the dendrogram corresponding to the hierarchical grouping of the estimators where this characteristic is displayed.

**Figure 9:**
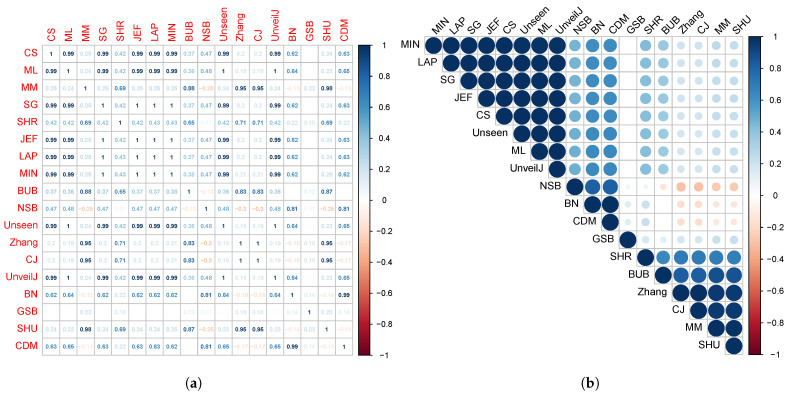
Correlation matrix between the estimators for random samples of bytes. The correlation is represented based on its numerical value (**a**) and the intensity of the color (**b**).

For bit samples, the behavior between all estimators is much more similar. However, a similar correlation analysis was performed. In Figure 9, the Kendall correlation coefficient values between the estimates for uniformly distributed samples of bits can be observed.

Figure 9 shows a division into fewer subgroups than for byte samples; this confirms no significant distinction in terms of bias in this scenario between the estimators. However, when applying the full link agglomerative method with correlation-based distance, it is obtained that it identifies four groups of estimators:

1. { NSB, CDM, BN},
2. { ML, SG, MIN, JEF, LAP, UnveilJ, CS, Unseen},
3. { GSB}.
4. { SHU, MM, CJ, Zhang, BUB, SHR}.

**Figure 10:**
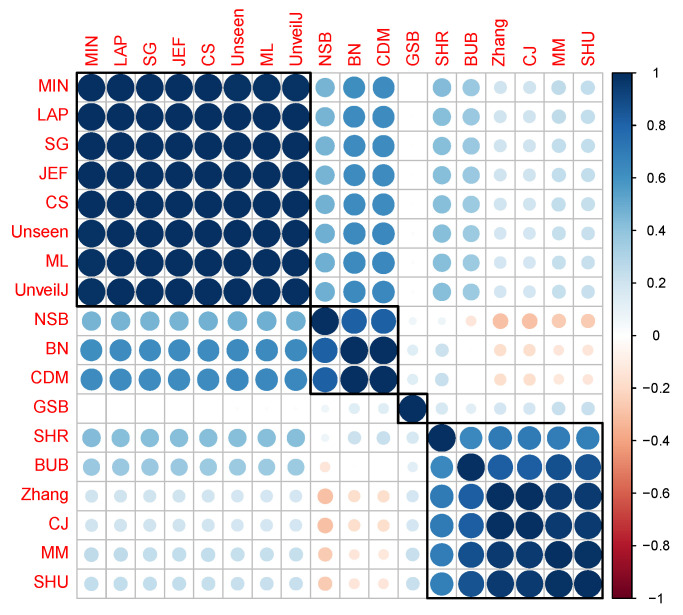
Clustering of estimators in uniformly distributed bit samples using the full link agglomerative method.

Figure 10 confirms the discussion raised about four of the estimators (CJ, SHU, MM, Zhang) with less bias in uniformly distributed samples of bits and, in turn, shows that although the average of $\hat{H}$ for the GSB estimator has satisfactory behavior, the values of this estimator are far from the behavior of the estimators of this group. This is the consequence of its high variance value. This case illustrates how the observation’s average can hide inappropriate behaviors and the need to use various statistical tools together.

**Figure 11:**
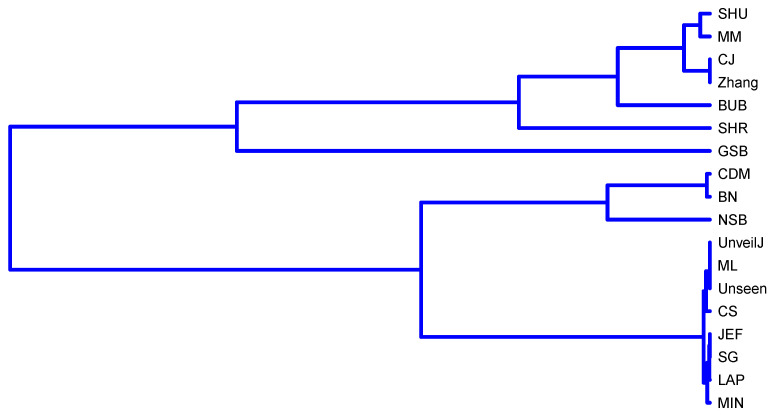
Dendrogram corresponding to the hierarchical grouping of the estimators for uniformly distributed samples of bits.

The SHR and BUB estimators are not displayed in Figure 1 within the estimator’s group with the best bias behavior. However, the agglomerative method detects that these two estimator’s values have a trajectory more similar to this group and superior to the other estimators. The relationship between the Bayesian estimators JEF, SG, MIN, and LAP is highlighted, which can be seen in Figure 11. Highlighting the relationship mentioned on the SG and JEF estimators in this scenario in Section 3.1.

The estimators {SHU,MM,CJ,Zhang,BUB,SHR} have similar behavior. It would be of interest to compare their efficiency because, in applications that require an extensive estimation of entropy, it is possible to evaluate using the most efficient of all of them, seeking a balance between effectiveness and efficiency. This comparison is left as an open problem for future work as it requires a unique own implementation that includes all these estimators.

## 5. Conclusions

In this work, a comparison was made between 18 estimators of entropy in short uniformly distributed samples of bytes and bits, based on their bias, variance, and mean square error. It was concluded that to estimate entropy in short uniformly distributed samples of bytes, the SHR estimator will be the most effective, while for bit samples, it is proposed to use the MM and SHR estimators. To estimate the entropy of random samples of bytes and bits, even considering the regime in short samples, based on the results obtained in this work, it is recommended to use the SHR estimator. The SHR estimator was the estimator with the least bias in random samples of bytes and the one with the highest convergence towards a mean square error equal to 0. For random samples of bits, it is within the set with the least bias, and within these, it is the estimator with the lowest dispersion in the values of the estimates with greater convergence towards an MSE = 0. It is noteworthy that the estimator is effective even for samples with the smallest sizes in both scenarios. This result is of great importance in cryptographic applications where scenarios with tiny samples often arise.

Additionally, the 18 estimators were grouped in clusters (different for each scenario) such that all the estimators of a cluster have similar effectiveness. This suggests that in applications that require intensive estimation of entropy, a selected estimator could be substituted for another correlated one, but of higher efficiency, which suggests comparing the efficiency of the estimators {CS,Unseen,C}∪{SHR} in uniformly distributed samples of bytes and the estimators {SHU,MM,CJ,Zhang,BUB,SHR} in uniformly distributed samples of bits, which will be addressed in future works.

## Figures and Tables

**Table 1:** Description of entropy estimators.

Known as	Notation	Estimator	
Miller-Madow correction [25]	MM	${\hat{H}}^{MM}=hatH^{ML}+\frac{m-1}{2n}$, with *m* the number of x∈K such that $y_{j}>0$	
Jackknife [44]	UnveilJ	${\hat{H}}^{JN\;}=n{\hat{H}}^{ML}-\frac{n-1}{n}{\hat{H}}_{-i}^{ML}$, donde ${\hat{H}}_{-i}^{ML}$ is the entropy of the sample original without the *i*-th symbol	
Best Upper Bound [4]	BUB	${\hat{H}}^{\mathrm{BUB}}=-\sum_{i=0}^{n\;}a_{i}h_{i}$, where $h_{i}=\sum_{x=1}^{k}1_{\left[y_{x}=i\right]}$ and $a_{i}=-\frac{i}{n}\log\frac{i}{n}+\left(\frac{1-\frac{i}{n}}{2n}\right)$	
Grassberger [41]	GSB	${\hat{H}}^{\mathrm{GSB}}=\log n-\frac{1}{n}\sum_{x=1}^{k}y_{x}\left(\psi \left(y_{x}\right)+{\left(-1\right)}^{y_{x}}\int_{0}^{1}\frac{t^{y_{x}-1}}{t+1}\mathrm{dt}\right)$ , where ψ(·) is the digamma function	
Schürmann [42]	SHU	${\hat{H}}^{\mathrm{SHU}}=\psi \left(n\right)-\frac{1}{n}\sum_{x=1}^{k}y_{x}\left(\psi \left(y_{x}\right)+{\left(-1\right)}^{y_{x}}\int_{0}^{\frac{1}{\xi }-1}\frac{t^{y_{x}-1}}{1+t}\mathrm{dt}\right)$	
Chao-Chen [28]	CS	${\hat{H}}^{\mathrm{CS}}=-\sum_{x\in K}^{\;}\frac{{\hat{p}}_{x}^{CS}\;{\log_{2}\hat{p}}_{x}^{\mathrm{CS}}}{1-{\left(1-{\hat{p}}_{x}^{\mathrm{CS}}\right)}^{n}}$, where ${\hat{p}}_{x}^{\mathrm{CS}}=\left(1-\frac{m}{n}\right){\hat{p}}_{x}^{ML}$	
James-Stein [32]	SHR	${\hat{H}}^{\mathrm{SHR}}=-\sum_{x\in K}^{\;}{\hat{p}}_{x}^{SHR}\;{\log_{2}\hat{p}}_{x}^{SHR}$, where ${\hat{p}}_{x}^{\mathrm{SHR}}=\hat{\lambda }t_{x}+\left(1-\hat{\lambda }\right){\hat{p}}_{x}^{ML}$ with $\hat{\lambda }=\frac{1-\sum_{x=1}^{k}\left({\hat{p}}_{x}^{ML}\right){}^{2}}{\left(n-1\right)\sum_{x=1}^{k}(t_{x}-{{\hat{p}}_{x}^{ML})}^{2}}$ and $t_{x}$ = 1/k	
Bonachela [40]	BN	$H^{\mathrm{BN}}=\frac{1}{n+2}\sum_{x=1}^{k}\left[\left(y_{x}+1\right)\sum_{j=y_{x}+2}^{n+2}\frac{1}{j}\right]$	
Zhang [34]	Zhang	${\hat{H}}^{\mathrm{Zhang}}=\sum_{v=1}^{n-1}\frac{1}{v}Z_{v}$, where $Z_{v}=\frac{n^{v+1}\left[n-\left(v+1\right)\right]!}{n!}\sum_{x\in K}^{}\left[{\hat{p}}_{x}\prod_{i=0}^{v-1}1-{\hat{p}}_{x}-\frac{i}{n}\right]$	
Chao-Wang-Jost [43]	CWJ	${\hat{H}}^{\mathrm{CWJ}}=\sum_{{1\leq y}_{x}\leq n-1}^{\;}\frac{y_{x}}{n}\left(\sum_{k=y_{x}}^{n-1}\frac{1}{k}\right)+$ $\frac{f_{1}}{n}{\left(1-A\right)}^{-n+1}\left\{-\log A-\sum_{r=1}^{n-1}\frac{1}{r}{\left(1-A\right)}^{r}\right\}$ with $A=\left\{\begin{array}{c} \frac{2f_{2}}{\left[\left(n-1\right)f_{1}+2f_{2}\right]}\;\;\;\;\;\;\;\mathrm{if}f_{2}>0\\ \frac{2}{\left[\left(n-1\right)\left(f_{1}-1\right)+2\right]}\;\;\;\;\;\;\;\;\;\;\;\;\mathrm{if}f_{2}=0,\;f_{1}\neq 0\;\\ 1\;\;\;\;\;\;\;\;\;\;\;\;\;\;\;\;\;\;\;\;\;\;\;\;\;\;\;\;\;\;\;\;\;\;\;\;\;\;\;\;\;\;\;\;\;\;\;\mathrm{if}\;\;f_{2}=\;f_{1}=0, \end{array}\right.\;$ where $f_{1}$ denote the number of singletons and $f_{2}$ denote the number of doubletons in the sample.	
Jeffrey [30]	JEF	${\hat{H}}^{\mathrm{Bayes}}=-\sum_{x\in K}^{\;}{\hat{p}}_{x}^{\mathrm{Bayes}}\;{\log_{2}\hat{p}}_{x}^{\mathrm{Bayes}}$, where ${\hat{p}}_{x}^{\mathrm{Bayes}}=\;\frac{y_{x}+a_{x}}{n+A}$ with $A=\sum_{x=1}^{k}a_{x}$	$a_{x}=1/2$
Laplace [29]	LAP		$a_{x}=1$
Schürmann- Grassberger [27]	SG		$a_{x}=1/k$
Minimax prior [31]	MIN		$a_{x}=\sqrt{n}/k$
NSB [26]	NSB	$H^{\mathrm{NSB}}=\frac{\int_{}^{}p\left(\xi ,n\right)H_{\beta }^{m}\left(n\right)d\xi }{\int_{}^{}p\left(\xi ,n\right)d\xi }$, where $p\left(\xi ,n\right)=\frac{\Gamma [k\beta (\xi )]}{\Gamma [n+k\beta (\xi )]}\prod_{x\in K}^{}\frac{\Gamma [y_{x}+\;\beta \left(\xi \right)]}{\Gamma [\beta \left(\xi \right)]}$, with $\xi =\psi_{0}\left(k\beta +1\right)-\psi_{0}\left(\beta +1\right)$, $\psi_{m}\left(x\right)={\left(d/\mathrm{dx}\right)}^{m+1}\;\log_{2}\Gamma \left(x\right)$ and $H_{\beta }^{m}\left(n\right)$ is the expectation value of the *m*-th entropy moment at fixed β; exact expression for m=1,2 is given in [49].	
CDM [45]	CDM	$H^{CDM}=\psi_{0}\left(N+a+1\right)-\sum_{x=1}^{K}\frac{y_{x}+a{\hat{\mu }}_{x}}{N+a}\left(y_{x}+a{\hat{\mu }}_{x}+1\right)$ where ${\hat{\mu }}_{x}={\hat{p}}_{x}^{ML}$	
Unseen [33]	Unseen	The authors propose to compute its value algorithmically.	

**Table 2:** Estimated mean of estimates for byte samples.

Estimators	Sample Sizes											
	8	16	32	64	128	256	512	1024	2048	4096	8192	16,384
CS	6.142	7.635	8.251	8.126	8.064	8.099	8.116	8.003	7.914	7.940	7.963	7.974
ML	2.969	3.943	4.880	5.768	6.550	7.178	7.591	7.809	7.908	7.955	7.977	7.989
MM	3.590	4.598	5.536	6.397	7.113	7.633	7.902	7.985	7.998	7.999	8.000	8.000
SG	3.999	4.470	5.136	5.884	6.598	7.194	7.595	7.809	7.908	7.955	7.977	7.989
SHR	7.940	7.971	7.982	7.991	7.995	7.998	7.999	7.999	8.000	8.000	8.000	8.000
JEF	7.947	7.902	7.834	7.751	7.690	7.696	7.767	7.854	7.919	7.957	7.978	7.989
LAP	7.983	7.969	7.943	7.904	7.859	7.832	7.842	7.883	7.928	7.960	7.979	7.989
MIN	5.048	5.385	5.835	6.357	6.875	7.326	7.643	7.823	7.912	7.956	7.978	7.989
BUB	7.008	7.021	7.476	7.732	7.868	8.061	7.896	7.971	7.983	7.985	7.985	7.985
NSB	6.427	7.065	7.474	7.726	7.855	7.922	7.953	7.969	7.977	7.981	7.983	7.984
Unseen	5.202	6.751	7.739	7.925	7.945	7.957	7.950	7.948	7.953	7.960	7.966	7.971
Zhang	3.690	4.696	5.627	6.478	7.178	7.674	7.916	7.979	7.985	7.985	7.985	7.985
CJ	5.349	7.028	8.089	8.093	8.004	7.996	7.987	7.985	7.985	7.985	7.985	7.985
UnveilJ	3.036	4.124	5.316	6.645	7.749	7.95	7.855	7.832	7.894	7.940	7.963	7.974
BN	2.963	4.433	5.815	6.981	7.726	7.889	7.41	6.662	6.115	5.829	5.686	5.616
GSB	4.646	5.613	6.464	7.182	7.670	7.921	7.981	7.985	7.985	7.985	7.985	7.985
SHU	4.347	5.329	6.210	6.978	7.541	7.868	7.972	7.985	7.985	7.985	7.985	7.985
CDM	6.165	6.908	7.391	7.689	7.844	7.922	7.961	7.981	7.990	7.995	7.998	7.999

**Table 3:** The bias of the estimates for the byte samples.

Estimator	8	16	32	64	128	256	512	1024	2048	4096	8192	16,384
CS	1.858	0.365	−0.251	−0.126	−0.064	−0.099	−0.116	−0.003	0.086	0.060	0.037	0.026
ML	5.031	4.057	3.120	2.232	1.450	0.822	0.409	0.191	0.092	0.045	0.023	0.011
MM	4.410	3.402	2.464	1.603	0.887	0.367	0.098	0.015	0.002	0.001	0.000	0.000
SG	4.001	3.530	2.864	2.116	1.402	0.806	0.405	0.191	0.092	0.045	0.023	0.011
SHR	0.060	0.029	0.018	0.009	0.005	0.002	0.001	0.001	0.000	0.000	0.000	0.000
JEF	0.053	0.098	0.166	0.249	0.310	0.304	0.233	0.146	0.081	0.043	0.022	0.011
LAP	0.017	0.031	0.057	0.096	0.141	0.168	0.158	0.117	0.072	0.040	0.021	0.011
MIN	2.952	2.615	2.165	1.643	1.125	0.674	0.357	0.177	0.088	0.044	0.022	0.011
BUB	0.992	0.979	0.524	0.268	0.132	-0.061	0.104	0.029	0.017	0.015	0.015	0.015
NSB	1.573	0.935	0.526	0.274	0.145	0.078	0.047	0.031	0.023	0.019	0.017	0.016
Unseen	2.798	1.249	0.261	0.075	0.055	0.043	0.050	0.052	0.047	0.040	0.034	0.029
Zhang	4.310	3.304	2.373	1.522	0.822	0.326	0.084	0.021	0.015	0.015	0.015	0.015
CJ	2.651	0.972	−0.089	−0.093	−0.004	0.004	0.013	0.015	0.015	0.015	0.015	0.015
UnveilJ	4.964	3.876	2.684	1.355	0.251	0.050	0.145	0.168	0.106	0.060	0.037	0.026
BN	5.037	3.567	2.185	1.019	0.274	0.111	0.590	1.338	1.885	2.171	2.314	2.384
GSB	3.354	2.387	1.536	0.818	0.330	0.079	0.019	0.015	0.015	0.015	0.015	0.015
SHU	3.653	2.671	1.79	1.022	0.459	0.132	0.028	0.015	0.015	0.015	0.015	0.015
CDM	1.835	1.092	0.609	0.311	0.156	0.078	0.039	0.019	0.010	0.005	0.002	0.001

**Table 4:** Estimated mean of the estimates for the bit samples.

Estimator	Sample Sizes											
	8	16	32	64	128	256	512	1024	2048	4096	8192	16,384
CS	0.935	0.95	0.975	0.987	0.993	0.995	0.997	0.997	0.998	0.998	0.998	0.998
ML	0.895	0.951	0.977	0.989	0.995	0.997	0.998	0.999	1.000	1.000	1.000	1.000
MM	0.984	0.996	0.999	1.000	1.000	1.000	1.000	1.000	1.000	1.000	1.000	1.000
SG	0.919	0.956	0.978	0.989	0.995	0.997	0.998	0.999	1.000	1.000	1.000	1.000
SHR	0.952	0.981	0.992	0.996	0.998	0.999	0.999	1.000	1.000	1.000	1.000	1.000
JEF	0.919	0.956	0.978	0.989	0.995	0.997	0.998	0.999	1.000	1.000	1.000	1.000
LAP	0.935	0.961	0.979	0.99	0.995	0.997	0.998	0.999	1.000	1.000	1.000	1.000
MIN	0.945	0.969	0.983	0.991	0.995	0.998	0.999	0.999	1.000	1.000	1.000	1.000
BUB	0.934	0.978	0.997	0.998	0.998	0.998	0.998	0.998	0.998	0.998	0.998	0.998
NSB	0.872	0.929	0.962	0.979	0.988	0.993	0.995	0.997	0.997	0.998	0.998	0.998
Unseen	0.894	0.949	0.975	0.987	0.993	0.995	0.997	0.997	0.998	0.998	0.998	0.998
Zhang	0.989	0.995	0.998	0.998	0.998	0.998	0.998	0.998	0.998	0.998	0.998	0.998
CJ	0.989	0.995	0.998	0.998	0.998	0.998	0.998	0.998	0.998	0.998	0.998	0.998
UnveilJ	0.893	0.949	0.975	0.987	0.993	0.995	0.997	0.997	0.998	0.998	0.998	0.998
BN	0.862	0.921	0.957	0.977	0.987	0.993	0.995	0.997	0.997	0.998	0.998	0.998
GSB	0.990	0.993	1.000	0.998	0.998	0.998	0.998	0.998	0.998	0.998	0.998	0.998
SHU	0.990	0.995	0.998	0.998	0.998	0.998	0.998	0.998	0.998	0.998	0.998	0.998
CDM	0.847	0.919	0.959	0.979	0.990	0.995	0.997	0.999	0.999	1.000	1.000	1.000

**Table 5:** Estimation bias for bit samples.

Estimator	8	16	32	64	128	256	512	1024	2048	4096	8192	16,384
CS	0.065	0.05	0.025	0.013	0.007	0.005	0.003	0.003	0.002	0.002	0.002	0.002
ML	0.105	0.049	0.023	0.011	0.005	0.003	0.002	0.001	0.000	0.000	0.000	0.000
MM	0.016	0.004	0.001	0.000	0.000	0.000	0.000	0.000	0.000	0.000	0.000	0.000
SG	0.081	0.044	0.022	0.011	0.005	0.003	0.002	0.001	0.000	0.000	0.000	0.000
SHR	0.048	0.019	0.008	0.004	0.002	0.001	0.001	0.000	0.000	0.000	0.000	0.000
JEF	0.081	0.044	0.022	0.011	0.005	0.003	0.002	0.001	0.000	0.000	0.000	0.000
LAP	0.065	0.039	0.021	0.01	0.005	0.003	0.002	0.001	0.000	0.000	0.000	0.000
MIN	0.055	0.031	0.017	0.009	0.005	0.002	0.001	0.001	0.000	0.000	0.000	0.000
BUB	0.066	0.022	0.003	0.002	0.002	0.002	0.002	0.002	0.002	0.002	0.002	0.002
NSB	0.128	0.071	0.038	0.021	0.012	0.007	0.005	0.003	0.003	0.002	0.002	0.002
Unseen	0.106	0.051	0.025	0.013	0.007	0.005	0.003	0.003	0.002	0.002	0.002	0.002
Zhang	0.011	0.005	0.002	0.002	0.002	0.002	0.002	0.002	0.002	0.002	0.002	0.002
CJ	0.011	0.005	0.002	0.002	0.002	0.002	0.002	0.002	0.002	0.002	0.002	0.002
UnveilJ	0.107	0.051	0.025	0.013	0.007	0.005	0.003	0.003	0.002	0.002	0.002	0.002
BN	0.138	0.079	0.043	0.023	0.013	0.007	0.005	0.003	0.003	0.002	0.002	0.002
GSB	0.01	0.007	0.000	0.002	0.002	0.002	0.002	0.002	0.002	0.002	0.002	0.002
SHU	0.01	0.005	0.002	0.002	0.002	0.002	0.002	0.002	0.002	0.002	0.002	0.002
CDM	0.153	0.081	0.041	0.021	0.01	0.005	0.003	0.001	0.001	0.000	0.000	0.000

**Table 6:** MSE estimates for byte samples.

Estimator	8	16	32	64	128	256	512	1024	2048	4096	8192	16,384
CS	3.722	0.716	1.024	0.312	0.054	0.022	0.017	0.001	0.007	0.004	0.001	0.001
ML	25.315	16.469	9.744	4.989	2.106	0.678	0.168	0.037	0.009	0.002	0.001	0.000
MM	19.466	11.583	6.085	2.581	0.793	0.139	0.011	0.001	0.000	0.000	0.000	0.000
SG	16.012	12.465	8.208	4.483	1.971	0.653	0.165	0.037	0.009	0.002	0.001	0.000
SHR	0.041	0.005	0.002	0.001	0.000	0.000	0.000	0.000	0.000	0.000	0.000	0.000
JEF	0.003	0.01	0.028	0.062	0.097	0.093	0.055	0.021	0.007	0.002	0.000	0.000
LAP	0.000	0.001	0.003	0.009	0.02	0.028	0.025	0.014	0.005	0.002	0.000	0.000
MIN	8.719	6.841	4.693	2.704	1.268	0.456	0.129	0.032	0.008	0.002	0.000	0.000
BUB	1.100	1.043	0.39	0.125	0.036	0.013	0.012	0.001	0.000	0.000	0.000	0.000
NSB	2.622	0.944	0.315	0.087	0.025	0.007	0.002	0.001	0.001	0.000	0.000	0.000
Unseen	8.000	1.859	0.71	0.328	0.079	0.018	0.006	0.004	0.003	0.002	0.001	0.001
Zhang	18.595	10.93	5.645	2.329	0.684	0.111	0.009	0.001	0.000	0.000	0.000	0.000
CJ	7.134	1.129	0.68	0.349	0.061	0.012	0.002	0.001	0.000	0.000	0.000	0.000
UnveilJ	24.658	15.051	7.246	1.929	0.092	0.023	0.024	0.028	0.011	0.004	0.001	0.001
BN	25.391	12.744	4.811	1.08	0.11	0.033	0.354	1.789	3.554	4.715	5.353	5.685
GSB	11.309	5.745	2.406	0.706	0.131	0.015	0.003	0.001	0.000	0.000	0.000	0.000
SHU	13.386	7.164	3.233	1.07	0.227	0.025	0.003	0.001	0.000	0.000	0.000	0.000
CDM	3.498	1.255	0.404	0.108	0.027	0.007	0.002	0.000	0.000	0.000	0.000	0.000

**Table 7:** MSE estimates for bit samples.

Estimator	8	16	32	64	128	256	512	1024	2048	4096	8192	16,384
CS	0.016	0.007	0.002	0.000	0.000	0.000	0.000	0.000	0.000	0.000	0.000	0.000
ML	0.033	0.008	0.002	0.000	0.000	0.000	0.000	0.000	0.000	0.000	0.000	0.000
MM	0.024	0.005	0.001	0.000	0.000	0.000	0.000	0.000	0.000	0.000	0.000	0.000
SG	0.019	0.006	0.001	0.000	0.000	0.000	0.000	0.000	0.000	0.000	0.000	0.000
SHR	0.019	0.003	0.001	0.000	0.000	0.000	0.000	0.000	0.000	0.000	0.000	0.000
JEF	0.019	0.006	0.001	0.000	0.000	0.000	0.000	0.000	0.000	0.000	0.000	0.000
LAP	0.012	0.005	0.001	0.000	0.000	0.000	0.000	0.000	0.000	0.000	0.000	0.000
MIN	0.008	0.003	0.001	0.000	0.000	0.000	0.000	0.000	0.000	0.000	0.000	0.000
BUB	0.026	0.007	0.001	0.000	0.000	0.000	0.000	0.000	0.000	0.000	0.000	0.000
NSB	0.028	0.008	0.002	0.001	0.000	0.000	0.000	0.000	0.000	0.000	0.000	0.000
Unseen	0.033	0.008	0.002	0.000	0.000	0.000	0.000	0.000	0.000	0.000	0.000	0.000
Zhang	0.024	0.005	0.001	0.000	0.000	0.000	0.000	0.000	0.000	0.000	0.000	0.000
CJ	0.024	0.005	0.001	0.000	0.000	0.000	0.000	0.000	0.000	0.000	0.000	0.000
UnveilJ	0.034	0.008	0.002	0.000	0.000	0.000	0.000	0.000	0.000	0.000	0.000	0.000
BN	0.032	0.009	0.003	0.001	0.000	0.000	0.000	0.000	0.000	0.000	0.000	0.000
GSB	0.063	0.015	0.003	0.001	0.000	0.000	0.000	0.000	0.000	0.000	0.000	0.000
SHU	0.024	0.005	0.001	0.000	0.000	0.000	0.000	0.000	0.000	0.000	0.000	0.000
CDM	0.041	0.011	0.003	0.001	0.000	0.000	0.000	0.000	0.000	0.000	0.000	0.000

## Data Availability

Not applicable.

## References

[1] Cover T.M., Thomas J.A. (2006). Elements of Information Theory.

[2] Verdú S. (2019). Empil Estimation of Information Measures: A Literature guide. Entropy.

[3] Vu V.Q., Yu B., Kass R.E. (2007). Coverage-adjusted entropy estimation. Stat. Med..

[4] Paninski L. (2003). Estimation of entropy and mutual information. Neural Comput..

[5] Antos A., Kontoyiannis I. (2001). Convergence properties of functional estimates for discrete distributions. Random Struct. Algorithms.

[6] Archer E., Park I.M., Pillow J.W. (2014). Bayesian entropy estimation for countable discrete distributions. J. Mach. Learn. Res..

[7] Timme N.M., Lapish C. (2018). A tutorial for information theory in neuroscience. eNeuro.

[8] Sechidis K., Azzimonti L., Pocock A., Corani G., Weatherall J., Brown G. (2019). Efficient feature selection using shrinkage estimators. Mach. Learn..

[9] Choudhury P., Kumar K.R., Nandi S., Athithan G. (2020). An empirical approach towards characterization of encrypted and unencrypted VoIP traffic. Multimed. Tools Appl..

[10] Zhang Y., Fu H., Knill E. (2020). Efficient randomness certification by quantum probability estimation. Phys. Rev. Res..

[11] Meyer P.E., Lafitte F., Bontempi G. (2008). Minet: A r/bioconductor package for inferring large transcriptional networks using mutual information. BMC Bioinform..

[12] Kurt Z., Aydin N., Altay G. (2016). Comprehensive review of association estimators for the inference of gene networks. Turk. J. Electr. Eng. Comput. Sci..

[13] Schulman J.S. (2019). Entropy: An Essential Component of Cryptographic Security. J. Cybersecur. Aware. Educ..

[14] Dai S., Guo D. (2015). Comparing security notions of secret sharing schemes. Entropy.

[15] Austrin P., Chung K.M., Mahmoody M., Pass R., Seth K. (2017). On the Impossibility of Cryptography with Tamperable Randomness. Algorithmica.

[16] Yasser I., Mohamed M.A., Samra A.S., Khalifa F. (2020). A chaotic-based encryption/decryption framework for secure multimedia communications. Entropy.

[17] Lu Q., Zhu C., Deng X. (2020). An Efficient Image Encryption Scheme Based on the LSS Chaotic Map and Single S-Box. IEEE Access.

[18] Knuth D. (1981). The Art of Computer Programming: Volume 2, Seminumerical Algoritms.

[19] (2011). Pseudorandom Number Sequence Test Program. http://www.fourmilab.ch/random/.

[20] (1995). Marsaglia, George; The Marsaglia Random Number CDROM Including the Diehard Battery of Tests of Randomness; Natl. Sci. Found. (Grants DMS-8807976 DMS-9206972). http://stat.fsu.edu/pub/diehard/.

[21] Rukhin A., Soto J., Nechvatal J., Miles S., Barker E., Leigh S., Levenson M., Vangel M., Banks D., Heckert A. (2010). SP800-22: A Statistical Test Suite for Random and Pseudorandom Number Generators for Cryptographic Applications. http://csrc.nist.gov/groups/ST/toolkit/rng/documents/SP800-22rev1a.pdf.

[22] L’ecuyer P., Simard R. (2007). TestU01: A C library for empirical testing of random number generators. ACM Trans. Math. Softw..

[23] Madarro-Capó E.J., Legón-Pérez C.M., Rojas O., Sosa-Gómez G., Socorro-Llanes R. (2020). Bit independence criterion extended to stream ciphers. Appl. Sci..

[24] Madarro Capó E.J., Cuellar O.J., Legón Pérez C.M., Gómez G.S. Evaluation of input—Output statistical dependence PRNGs by SAC. Proceedings of the 2016 International Conference on Software Process Improvement (CIMPS).

[25] Miller G. (1955). Note on the bias of information estimates. Inf. Theory Psychol. Probl. Methods.

[26] Nemenman I., Shafee F., Bialek W. (2001). Entropy and Inference, Revisited. arXiv.

[27] Schürmann T., Grassberger P. (1996). Entropy estimation of symbol sequences. Chaos.

[28] Chao A., Shen T.J. (2003). Nonparametric estimation of Shannon’s index of diversity when there are unseen species in sample. Environ. Ecol. Stat..

[29] Holste D., Große I., Herzel H. (1998). Bayes’ estimators of generalized entropies. J. Phys. A. Math. Gen..

[30] Krichevsky R.E., Trofimov V.K. (1981). The Performance of Universal Encoding. IEEE Trans. Inf. Theory.

[31] Trybula S. (1958). Some problems of simultaneous minimax estimation. Ann. Math. Stat..

[32] Hausser J., Strimmer K. (2009). Entropy inference and the james-stein estimator, with application to nonlinear gene association networks. J. Mach. Learn. Res..

[33] Valiant G., Valiant P. (2017). Estimating the unseen: Improved estimators for entropy and other properties. J. ACM.

[34] Zhang Z. (2012). Entropy estimation in Turing’s perspective. Neural Comput..

[35] Daub C.O., Steuer R., Selbig J., Kloska S. (2004). Estimating mutual information using B-spline functions—An improved similarity measure for analysing gene expression data. BMC Bioinform..

[36] Margolin A.A., Nemenman I., Basso K., Wiggins C., Stolovitzky G., Favera R.D., Califano A. (2006). ARACNE: An algorithm for the reconstruction of gene regulatory networks in a mammalian cellular context. BMC Bioinform..

[37] Van Huile M.M. (2005). Edgeworth approximation of multivariate differential entropy. Neural Comput..

[38] Vinck M., Battaglia F.P., Balakirsky V.B., Vinck A.J., Pennartz C. (2012). Estimation of the entropy on the basis of its polynomial representation. IEEE Int. Symp. Inf. Theory Proc..

[39] Kozachenko L.F., Leonenko N.N. (1987). Sample Estimate of the Entropy of a Random Vector. Probl. Inf. Transm..

[40] Bonachela J.A., Hinrichsen H., Mũoz M.A. (2008). Entropy estimates of small data sets. J. Phys. A Math. Theor..

[41] Grassberger P. (2003). Entropy estimates from insufficient samplings. arXiv.

[42] Schürmann T. (2004). Bias analysis in entropy estimation. J. Phys. A. Math. Gen..

[43] Chao A., Wang Y.T., Jost L. (2013). Entropy and the species accumulation curve: A novel entropy estimator via discovery rates of new species. Methods Ecol. Evol..

[44] Burnham K.P., Overton W.S. (1978). Estimation of the Size of a Closed Population when Capture Probabilities vary Among Animals. Biometrika.

[45] Archer E., Park I.M., Pillow J.W. (2013). Bayesian entropy estimation for binary spike train data using parametric prior knowledge. Adv. Neural Inf. Process. Syst..

[46] Valiant G., Valiant P. (2011). Estimating the unseen: An n/log(n)-sample estimator for entropy and support size, shown optimal via new CLTs. Proc. Annu. ACM Symp. Theory Comput..

[47] Nemenman I. (2011). Coincidences and estimation of entropies of random variables with large cardinalities. Entropy.

[48] Al-Omari A.I. (2015). New entropy estimators with smaller root mean squared error. J. Mod. Appl. Stat. Methods.

[49] Wolpert D.H., Wolf D.R. (1995). Estimating functions of probability distributions from a finite set of samples. Phys. Rev. E.

[50] Schürmann T. (2015). A note on entropy estimation. Neural Comput..

[51] de Matos Simoes R., Emmert-Streib F. (2012). Influence of Statistical Estimators on the Large-Scale Causal Inference of Regulatory Networks. Stat. Mach. Learn. Approaches Netw. Anal..

[52] Müller S. (2018). Linux Random Number Generator-A New Approach. http://www.chronox.de/lrng/doc/lrng.pdf.

[53] Marton K., Suciu A., Ignat I. (2010). Randomness in digital cryptography: A survey. Rom. J. Inf. Sci. Technol..

[54] Zhang Z., Grabchak M. (2014). Nonparametric estimation of Küllback-Leibler divergence. Neural Comput..

[55] GitHub—Simomarsili/ndd: Bayesian Entropy Estimation in Python—Via the Nemenman-Schafee-Bialek Algorithm. https://github.com/simomarsili/ndd.

[56] Marcon E., Herault B. (2015). entropart: An R package to measure and partition diversity. J. Stat. Softw..

[57] GitHub—Pillowlab/CDMentropy: Centered Dirichlet Mixture Entropy Estimator for Binary Data. https://github.com/pillowlab/CDMentropy.

[58] Rosenblad A. (2011). The Concise Encyclopedia of Statistics.

[59] Yim O., Ramdeen K.T. (2015). Hierarchical Cluster Analysis: Comparison of Three Linkage Measures and Application to Psychological Data. Quant. Methods Psychol..

[60] Ma X., Dhavala S. (2018). Hierarchical clustering with prior knowledge. arXiv.

